# Investigation of He’s Yang Chao recipe against oxidative stress-related mitophagy and pyroptosis to improve ovarian function

**DOI:** 10.3389/fendo.2023.1077315

**Published:** 2023-01-27

**Authors:** Chenyun Miao, Ying Zhao, Yun Chen, Ruye Wang, Ning Ren, Bixia Chen, Pingpei Dong, Qin Zhang

**Affiliations:** Department of Traditional Chinese Medical Gynecology, Hangzhou Hospital of TCM Affiliated to Zhejiang Chinese Medical University, Hangzhou, China

**Keywords:** oxidative stress, pyroptosis, inflammasome, primary ovarian insufficiency, traditional chinese medicine

## Abstract

**Background:**

Primary ovarian insufficiency (POI) is a common gynecological disease with serious ramifications including low pregnancy rate and low estrogen symptoms. Traditional Chinese medicine is regarded as an effective treatment for POI. However, the therapeutic mechanism of it is unclear.

**Methods:**

In this study, a mouse model of primary ovarian insufficiency was established by intraperitoneal injection of cyclophosphamide (CTX) and He’s Yang Chao Recipe (HSYC) concentrate was used for intragastric administration. Serum hormone levels (Anti-Müllerian Hormone, Estradiol, Progesterone, Luteinizing Hormone and Follicle Stimulating Hormone) and Oxidative Stress (OS) related products, superoxide dismutase (SOD), GSH-Px, and malondialdehyde (MDA) were measured by enzyme-linked immunosorbent assay. Pathological changes in ovarian tissue were evaluated by hematoxylin and eosin staining, and flow cytometry was used to determine reactive oxygen species content and mitochondrial membrane potential levels in granulosa cells. Mitochondrial distribution and morphology were investigated using immunofluorescence staining. The level of mitophagy was evaluated by LC3 immunofluorescence staining and autophagosome counts using electron microscopy. Western blotting and qPCR were used to detect the expression of proteins and genes related to mitophagy and the NLRP3 inflammasome.

**Results:**

After HSYC treatment, the ovarian damage was milder than in the CTX group. Compared with the CTX group; SOD, GSH-Px, and the total antioxidant capacity were significantly increased, while MDA and ROS were decreased in the HSYC treatment groups. Furthermore, mitochondrial distribution and membrane potential levels were improved after HSYC treatment compared to the CTX group. After the HSYC treatment, the LC3 fluorescent intensity and autophagosome counts were decreased. Similarly, mitophagy related markers PINK1, Parkin, LC3, and Beclin1 were decreased, while p62 was significantly increased, compared with the CTX groups. The mRNA and protein expression of NLRP3 inflammasome, NLRP3, caspase-1, GSDMD, IL-18, and IL-1β were significantly decreased in the HSYC treatment groups.

**Conclusion:**

This is the first study in molecular mechanisms underlying HSYC against granulosa cell injury in POI. HSYC protects ovaries from CTX-induced ovarian damage and oxidative stress. HSYC enhanced ovarian function in mice with primary ovarian insufficiency by inhibiting PINK1-Parkin mitophagy and NLRP3 inflammasome activation.

## 1 Introduction

Primary ovarian insufficiency (POI) leads to cessation of menstruation before the projected menopause age, causing amenorrhea or estrogen-deficiency symptoms. Currently, the definition of POI includes amenorrhea for at least four months and high follicle stimulating hormone (FSH) values of >25 mIU/mL (1). It is estimated that 1% of the global population is affected by POI before the age of 40 years, and 0.1% before the age of 30 (2). Infections, autoimmune diseases, iatrogenic operations, metabolic abnormalities, and environmental variables are underlying causes (3–5), however, the precise pathophysiology of POI remains unknown.

The use of traditional Chinese medicine (TCM) for the treatment of POI has attracted much attention owing to its therapeutic efficacy and low toxicity. He’s Yang Chao Recipe (HSYC) is derived from “Cong Rong Tu Si Wan”, a classic and renowned formula recorded in the traditional Chinese medicinal book Ji-Yin-Zong-Lu in the Ming Dynasty. HSYC primarily contains eight herbal drugs (**Table 1**). According to TCM theories, the basic pathogenesis of POI is related to deficiency of kidney essence (6).

**Table 1:** He’s Yang Chao recipe (HSYC) composition.

Chinese name	Scientific name	Family	Plant part	Crude herb (g)
Baishao	*Paeonia lactiflora* Pall	Paeoniaceae	Root	10
Tusizi	*Cuscuta chinensis* Lam	Convolvulaceae	Seeds	15
Roucongrong	*Cistanche salsa* (C.A. Mey.) G. Beck	Orobanchacea	Stem	15
Danggui	*Angelica sinensis* (Oliv.) Diels	Apiaceae	root	10
Fupenzi	*Rubus chingii* Hu	Rosaceae	fruit	15
Gegen	*Pueraria lobata* (Willd.) Ohwi	Leguminosae	root	12
Tiandong	*Asparagus cochinchinensis* (Lour.) Merr.	Asparagaceae	root	10
Baiziren	*Platycladus orientalis* (Linn.) Franco	Cupressaceae	seed	10

The primary function of HSYC is nourishing kidney and replenish essence. Cuscuta chinensis and Cistanche salsa can warm kidney yang, Rubus chingii, Pueraria lobata, and Asparagus cochinchinensis reinforce kidney ying. Paeonia lactiflora Pall and Angelica sinensis can enrich blood, and Platycladus orientalis can nourish the heart and tranquilize mind.Previous investigations indicates that HSYC has positive effects on ovarian reserve, oocyte quality, and embryo hatching potential (7, 8).

To help explore its mechanism of action, liquid chromatography-mass spectrometry was used to identify the main bioactive components of HSYC (8). These included: kaempferol, daidzein, astragalin, genistein, hyperin, rutin, gallic acid, acteoside, ellagic acid, quercetin, z-ligustilide, echinacoside, nicotiflorin, paeoniflorin, ferulic acid, apigenin, and puer. HSYC also has potential positive effects on ovarian function, egg quality and embryo development, and may be able to counteract the adverse effects of age and consecutive ovarian superovulation (8). However, the underlying mechanism of HSYC in POI is not well-studied.

Oxidative stress (OS) is closely related to several physiological functions during the female reproductive age, including follicular development, ovulation, implantation, follicle formation, luteolysis, and luteal maintenance of pregnancy; even beyond menopause (9–11). Under normal condition, oxides, such as reactive oxygen species (ROS) and reactive nitrogen species, and antioxidants including superoxide dismutase (SOD) and glutathione peroxidase (GSH-Px), restrict one another to maintain the redox homeostasis in the cell (12). Once the balance between these is disrupted, OS is established. Excessive ROS is the most important constituent of OS and can cause a range of oxidative damage to cellular components and organelles that in turn can impair cellular processes (13). POI development is linked to excessive accumulation of ROS in the ovaries, which accelerates ovarian cell senescence and decreases ovarian function (14).

Mitochondria are the primary source of ROS, they not only regulate the level of ROS produced but are also very sensitive to OS injury. ROS accumulation causes mitochondrial DNA damage, which further leads to mitochondrial dysfunction (15). When mitochondrial function is compromised, the mitochondrial stress response mechanism, also known as mitophagy, is initiated to prevent further damage and restore mitochondrial homeostasis (16, 17). Mitophagy activation *via* OS depends on the phosphatase and tensin homolog (PTEN)-induced putative kinase 1 (PINK1), whereas Parkin-mediated mitophagy relies on the superoxide anion. The direct generation of mitochondrial ROS production using mitochondrial target sensitizers has been demonstrated in various studies (18, 19). Additionally; pyroptosis, an inflammation-dependent programmed cell death, is a serious adverse outcome of OS (20). Pyroptosis commences with the activation of pathogen-associated molecular patterns (PAMPs) or damage-associated molecular patterns (DAMPs), the oligomerization of NLRP3, and the recruitment of ASC and caspase-1 which aggregate and form the NLRP3 inflammasome. Activated caspase-1 can then cleave pro-IL-1β, pro-IL-18, and the pore-forming protein gasdermin D, resulting in pyroptosis and the release of the pro-inflammatory cytokines IL-1β and IL-18 (21). The relationship between pyroptosis and ovarian insufficiency through the increased expression of NLRP3, IL-1β, and caspase-1 in ovarian granulosa cells (GCs) of POI patients has been previously reported (22).

**Figure 1:**
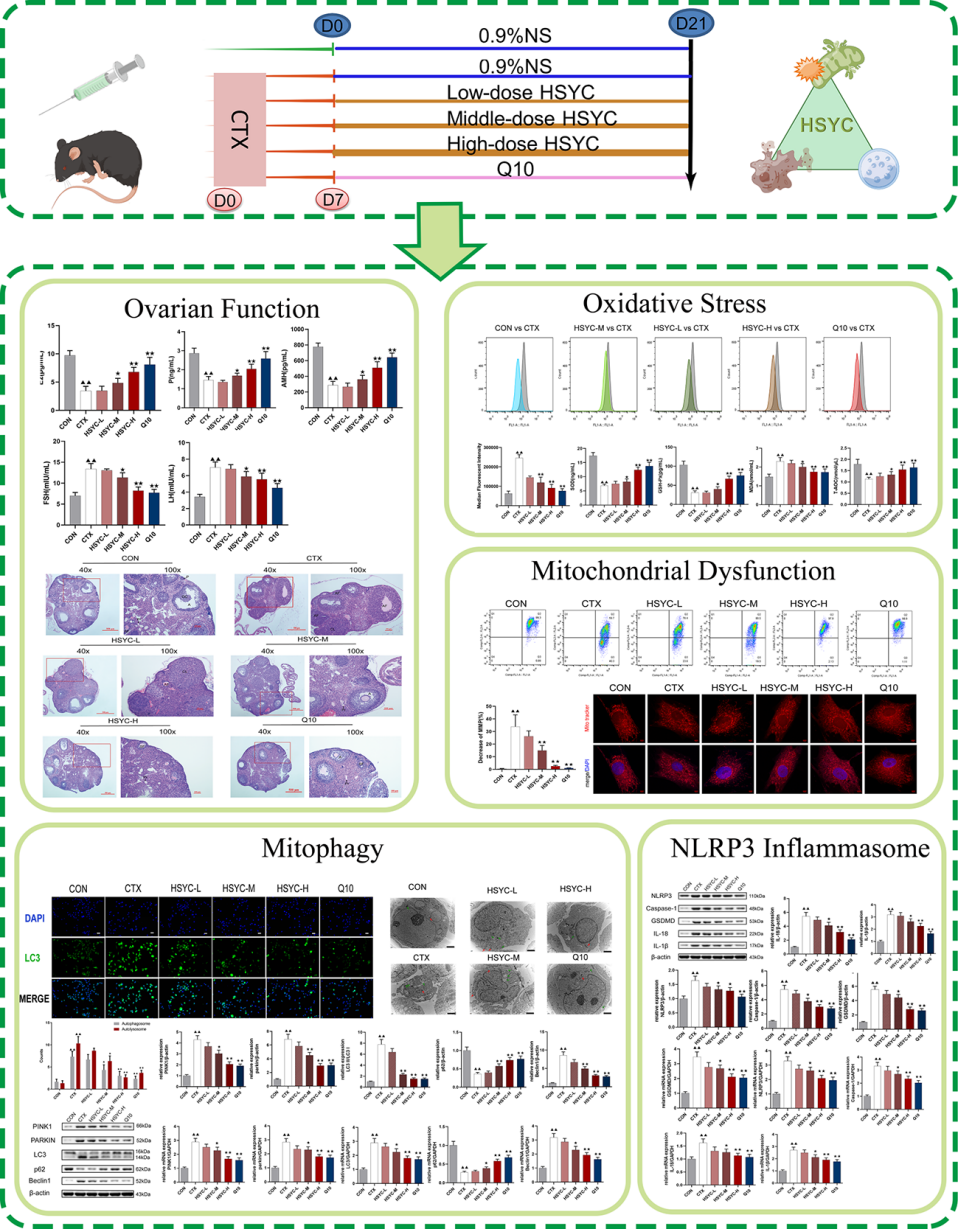
The flowchart of this study.

In this study, we investigated whether HSYC can affect OS, mitophagy, and pyroptosis in experimental POI models to determine its underlying mechanisms. **Figure 1** illustrates the workflow of this study.

## 2 Methods and material

### 2.1 HSYC preparation

The eight herbs used in HSYC (**Table 1**) were purchased from the Hangzhou Hospital of Traditional Chinese Medicine. After soaking, boiling with distilled water, concentrating to a thick slurry, and dried to powder format with a crude dose of 2.25 g/ml. The resulting HSYC powder was dissolved in water and used for murine intragastric administration.

### 2.2 Ethical statement

The research was conducted in accordance with all relevant ethical regulations. Institutional Animal Care, the Animal Experiment Center of Zhejiang Chinese Medical University, approved all the experimental procedures used in this study (Approval number: IACUC-20180402-02).

### 2.3 Animal models and treatment

Sixty C57/BL6 female mice aged 6–8 weeks were obtained from Shanghai Laboratory Animal Company (SLACCAS, license no. SCXK 2017-0005, Shanghai, China). Mice were raised under standard conditions for a week before the formal experiment in a barrier facility with stable specific-pathogen-free levels, temperatures, humidity, and light cycles in the Animal Experimental Research Center of Zhejiang Chinese Medical University (license no. SYXK 2021-0012, Zhejiang, China). Research was performed according to the Animal Research: Reporting of *In Vivo* Experiments (ARRIVE) guidelines. The 60 mice were randomly distributed into six groups: CON, CTX, HSYC-L, HSYC-M, HSYC-H, and Q10 groups. Based on published literature and our pre-experiment result, the POI model was injected intraperitoneally with cyclophosphamide (CTX) at 90 mg/kg (7), and the success rate was determined by observing the estrous cycle for 7 days. Once the POI model was established, the Q10 group were intragastrically administered coenzyme Q10 (Eisai, Tokyo, Japan) at a dose of 1.25 mg/kg, while mice in the HSYC-L, HSYC-M, and HSYC-H groups were given HSYC by intragastric administration at doses of 6 g/kg, 12 g/kg, and 25 g/kg at 8 AM daily, according to the conversion of human clinical dosage.

Mice in the CON and CTX groups were administered saline solution by gavage. Duration of treatment was 3 weeks. Within 48 hours of PMSG injection, CO_2_ inhalation was used to euthanize the mice and blood and ovaries were collected for subsequent use. Actually, the modeling success rate was about 70%, 30 mice being successfully established (n=6).

### 2.4 Primary granulosa cell extraction and culture

Fresh murine ovarian tissues were placed in a culture dish. F12 media was then added and the follicles were punctured with a sterile 1 mL syringe needle to release the GCs into the culture media. GCs were gently blown, filtered with a 200 mesh sieve, and centrifuged at room temperature for 3 min at 1000 ×g. The GCs were then transferred to a new sterile petri dish and incubated for 48 hours at 37°C with 5% CO_2_. The nonadherent cells were removed and the media changed every 72 h.

### 2.5 Enzyme-linked immunosorbent assay

Serum samples obtained before the mice were euthanized were centrifuged at room temperature for 20 min at 3000 ×g. Serum hormone levels, including anti-Müllerian hormone (AMH), estradiol (E2), progesterone (P), luteinizing hormone (LH), and FSH; and OS related products, SOD, GSH-Px, and malondialdehyde (MDA), were measured using ELISA kits (Meimian, Shanghai, China), according to the manufacturer’s instructions. These included Mouse E2 ELISA kit, Mouse LH kit, Mouse FSH kit, Mouse P kit, Mouse AMH kit, Mouse SOD kit, Mouse GSH-Px kit, and Mouse MDA kit.

### 2.6 Ferric reducing antioxidant potential assay

The total antioxidant capacity (T-AOC) of serum samples was also assessed. Serum samples, standards, and distilled water blank controls were prepared according to the T-AOC assay kit (Aidisheng, Jiangsu, China). The absorbance value was then determined at 590 nm in a CMaxPlus microplate reader (Molecular Devices, San Jose, CA, USA).

### 2.7 ROS and mitochondrial membrane potential flow cytometric assay

ROS content was determined by flow cytometry using 2’-7’-dichlorodihydrofluorescein diacetate (DCFH-DA) (Beyotime, Shanghai, China). DCFH-DA was diluted with serum-free medium at 1:1000, with a final concentration of 10 μmol/L. Diluted DCFH-DA (1 mL) was added to the cells of each group after removing the cell culture media and incubated at 37 °C for 20 min. Cells were then washed three times with serum-free cell culture solution and prepared for flow cytometric detection. GC mitochondrial membrane potential (MMP) levels were detected using the JC-1 assay kit (Beyotime). Cell culture media were removed in each group and 1 mL incomplete culture medium and 1 mL JC-1 working solution was added after washing with PBS. Negative and positive control groups were set according to the instructions. All groups were incubated at 37 °C in the dark for 20 min. GCs were then digested, collected, centrifuged, washed twice with JC-1 staining buffer, and resuspended before flow cytometry (C6, BD, USA) was performed.

### 2.8 Immunofluorescence staining

A 4% paraformaldehyde solution was used for fixation of the cells, 0.5% Triton X-100 for permeabilization, and 3% bovine serum albumin for blocking. Anti-LC3 primary antibody (Cell Signaling Technology, Danvers, MA, USA) was added, the solution was incubated overnight, and the secondary antibody was added and incubated in dark for 30 min. After staining the nucleus with 4’,6-diamidino-2-phenylindole, the sections were sealed with an anti-fluorescence quenching agent and visualized using a Ts2-FC fluorescence microscope (Nikon, Tokyo, Japan).

### 2.9 GCs mitochondrial morphology

Mito Tracker Red CMXRos (Beyotime, Shanghai, China) solution (200 µM) was added to the cell culture media at a ratio of 1:2000 to pre-incubate at 37 °C and subsequently, 500 µL diluted Mito Tracker Red CMXRos working solution was added to each well. Cell culture media was then infused with Hoechst solution, and incubated for 10 minutes without light. A Ts2-FC inverted fluorescence microscopy was used to visualize the mitochondrial morphology (Nikon).

### 2.10 Electron microscopy

The cells were collected and fixed in glutaraldehyde and by osmic acid, rinsed, gradient ethanol dehydrated, and embedding agent penetrated and sectioned. After staining with uranium acetate and lead citrate, the sections were visualized and images captured under transmission electron microscope (TEM) (H-7650, HITACHI, Tokyo, Japan).

### 2.11 Histopathology

Following paraffin embedding and sectioning (4 μm), ovarian tissues were dehydrated with xylene and ethanol, stained with hematoxylin and eosin, sealed and visualized under an Eclipse E100 microscope (Nikon). The ovarian structure was observed and the number of follicles at all levels were counted.

### 2.12 Real-time qualitative PCR

Following RNA extraction by the Trizol standard method (Thermo Fisher Scientific, Waltham, MA, USA), RNA was reverse transcribed in a thermocycler (Mastercycler; Eppendorf, Hamburg, Germany) using the cDNA reverse transcriptase kit (CWBiotech, Beijing, China), according to the manufacturer’s instructions. The cDNA was then amplified using the fluorescence-quantitative PCR kit (TAKARA, Shiga, Japan), according to the manufacturer’s instructions. The cycling conditions were as follows: 95 °C, 10 min; 95 °C, 15 s; 60 °C, 60 s; for 40 cycles. The reactions were performed using the CFX Connect Real-Time PCR Detection System (Bio-Rad, Hercules, CA, USA), and the gene expression levels were calculated by the 2^-△△CT^ method. The primer sequences (5′ to 3′) of target genes are shown in **Table 2**.

**Table 2:** Primer sequences of the target genes.

Gene	Forward primer	Reverse primer
PINK1	TGCTGAAACTGCCTTCCTATCA	CCGCTAGTTGAACATACAGGATG
Parkin	GCTTCCGAAGGTGTGTCAG	CGGGCATTGCTCTCAGTCA
LC3	GGCTACGGCTACTATCGCAC	GGAGAAGGTTTTGCGGTTGAAA
p62	ACAGCCCAAACGTGCAGTAA	CTGATGCGGAACTACATCTGAAT
Beclin1	ATGGAGGGGTCTAAGGCGTC	TGGGCTGTGGTAAGTAATGGA
NLRP3	ATTACCCGCCCGAGAAAGG	CATGAGTGTGGCTAGATCCAAG
Caspase-1	CGCCCTGTTGGAAAGGAACT	GCAAGACGTGTACGAGTGGT
GSDMD	ATGCCATCGGCCTTTGAGAAA	AGGCTGTCCACCGGAATGA
IL-18	AACACTGGCTGTTCCCACAA	TCCAGGTCTCCATTTTCTTCAGG
IL-1β	TTGAAGTTGACGGACCCCAA	TGTCCTGACCACTGTTGTTTC
GAPDH	TCAACGGCACAGTCAAGG	TGAGCCCTTCCACGATG

### 2.13 Western blotting

After addition of radioimmunoprecipitation assay lysis buffer (Beyotime), tissues were ground in a homogenizer and then lysed on ice for 1 h. The protein concentration was then determined using the BCA Kit (Solarbio, Beijing, China). The proteins were denatured with loading buffer at 100 °C for 5 min. Following protein gel electrophoresis, membrane transfer, blocking, and incubation of primary antibody and secondary antibody, photographic developer was added to the membrane and the band was developed in ECL chemiluminescence instrument with β-actin as internal reference. The antibodies as follows: anti-PINK1 (Affinity Biologicals, Ancaster, ON, Canada; DF7742; 1:1,000), anti-Parkin (Affinity Biologicals; AF0235; 1:1,000), anti-LC3 (Cell Signaling Technology; 4108s; 1:1,000), anti-p62 (Cell Signaling Technology; 8025s; 1:1,000), anti-Beclin1 (Affinity Biologicals; AF5128; 1:1,000), anti-NLRP3 (Affinity Biologicals; DF7438; 1:1,000), anti-GSDMD, anti-IL-18 (Affinity Biologicals; AF4012; 1:1,000), anti-caspase-1 (Proteintech, Rosemont, IL, USA; 22915-1-AP; 1:1,000), anti-IL-1β (Proteintech; 16806-1-AP; 1:1,000), and anti β-actin (Affinity Biologicals; AF7018; 1:2,000).

### 2.14 Statistical analyses

Study data are presented as means ± standard deviations (SD) and were analyzed statistically using one-way analysis of variance and Dunnett’s test. A statistically significant difference was defined as a *P*-value less than 0.05 (*P<0.05*), and *P*-value less than 0.01 (*P<0.01*) was considered highly significant.

## 3 Results

### 3.1 HSYC improved ovarian function in POI afflicted mice

**Figure 2:**
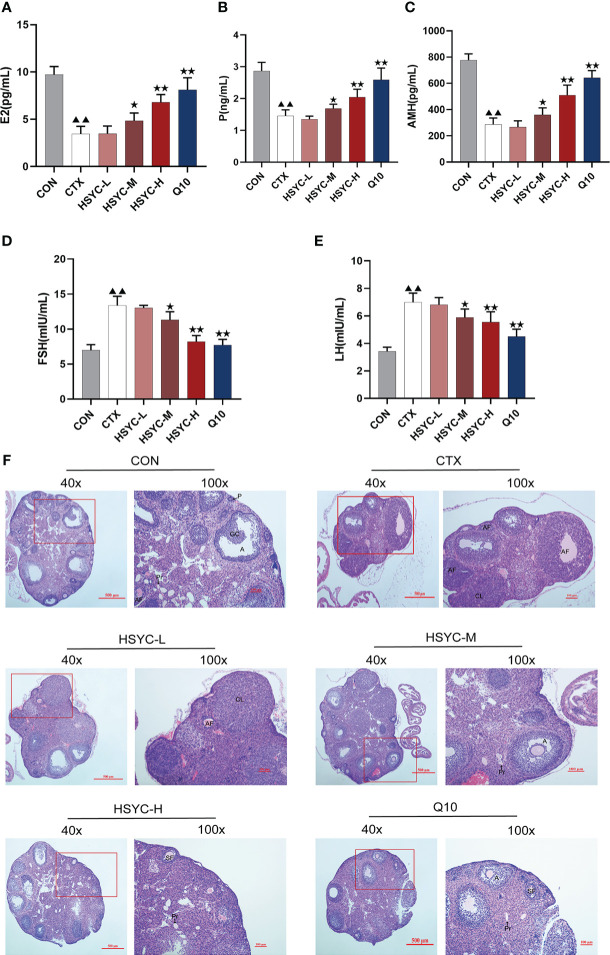
HSYC improved ovarian function in POI afflicted mice. **(A-E)** The level of serum estrogen, progesterone, anti-Müllerian hormone, follicle stimulating hormone and luteinizing hormone of mice in different groups. **(F)** Ovarian tissue sections with H&E staining in different groups. Histogram elements represent the mean ± SD (n=6). ^▲▲^ presents *P<0.01* compared to the CON group; ^★^presents *P < 0.05* compared to the CTX group; ^★★^presents *P < 0.01* compared to the CTX group. E2, estradiol; P, progesterone; AMH, anti-Müllerian hormone; FSH, follicle stimulating hormone; LH, luteinizing hormone.

Follicle development and sex hormone levels predict ovarian function. Compared with the CON group, the E2, P and AMH serum levels in the CTX group were significantly decreased (P<0.01); however, FSH and LH levels were higher than those the CON group. Following medium-dose HSYC, high-dose HSYC, or Q10 intervention; E2, P, and AMH were significantly increased (P<0.05 or P<0.01), while FSH and LH levels were decreased compared to the CTX group (P<0.05 or P<0.01) (**Figures 2A-E**).

The ovaries in the CON group were well-developed with visible follicles at all grades. In contrast, those of the CTX group showed atrophy, and the number of primary and secondary follicles was significantly reduced compared with the CON group (P<0.01). Furthermore, GC arrangement in mature follicles remained disturbed. Follicles of different stages and corpora lutea could be observed in all treatment groups, especially in HSYC-H and Q10 groups where the cumulus GCs in mature follicles were neatly arranged radially (**Figure 2F**). According to the follicle count results, the number of primary follicles was significantly higher in most of the treatment groups compared to the CTX group (P<0.05 or P<0.01); however, the number of primordial and mature follicles did not differ significantly between these groups (P≥0.05) (**Table 3**).

**Table 3:** Differential follicle count in the ovaries of each group.

Group	Primordial follicles	Primary follicles	Secondary follicles	Mature follicles
CON	8.83 ± 3.97	6.17 ± 1.17	4.5 ± 1.05	2.83 ± 0.98
CTX	7.67 ± 1.63	2.00 ± 0.89^▲▲^	2.67 ± 0.52^▲▲^	2.17 ± 0.75
HSYC-L	6.83 ± 1.72	2.50 ± 1.05	2.33 ± 0.52	2.33 ± 0.82
HSYC-M	7.50 ± 1.87	3.83 ± 1.47^★^	3.33 ± 1.21	2.83 ± 1.47
HSYC-H	8.67 ± 2.07	4.00 ± 1.10^★★^	3.17 ± 0.98	3 ± 1.26
Q10	8.33 ± 2.58	4.83 ± 0.75^★★^	2.83 ± 0.98	3.33 ± 1.51

^▲▲^presents P < 0.01 compared to the CON group; ^★^presents P<0.05 compared to the CTX group ^★★^presents P < 0.01 compared to the CTX group.

### 3.2 Identification of granulosa cells

As is shown in **Figure S1A, B**, the isolated GCs (mainly consists of GCs but not purified) grew and adhered the wall. After 24 hours of culture, GCs begin to adhere to the wall and proliferate. After 48h of culture, the cells proliferated significantly and grew vigorously. It can be seen under the microscope that the adherent cells are complete in shape, clear in edge, uniform in size, polygonal or spindle shaped, the nucleus is blue with oval shape and the cytoplasm is light pink by hematoxylin and eosin staining.

### 3.3 HSYC decreased OS of POI mice

**Figure 3:**
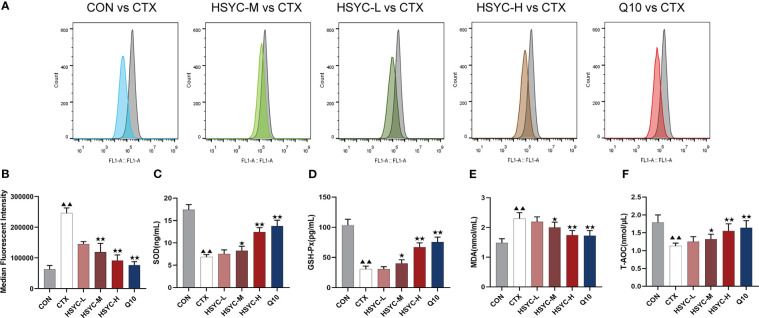
HSYC decreased oxidative stress of POI afflicted mice. **(A)** Flow cytometric analysis of ROS accumulation in ovarian granulosa cells of different groups, and histogram plots represents means with SD of median fluorescence intensity **(B)**. **(C-F)** the levels of serum superoxide dismutase, glutathione peroxidase, malondialdehyde and total antioxidant capacity of mice in different groups. Histogram elements represent the mean ± SD (n=6). ^▲▲^ presents *P< 0.01* compared to the CON group; ^★^presents *P < 0.05* compared to the CTX group; ^★★^presents *P<0.01* compared to the CTX group. SOD, superoxide dismutase; GSH-Px, glutathione peroxidase; MDA, malondialdehyde; T-AOC, total antioxidant capacity.

In the flow cytometry result, mean fluorescence intensity represented ROS expression. The ROS level was significantly increased in the CTX group compared to the CON group (*P<0.01*); while ROS production was inhibited in different treatment groups, including the HSYC-M, HSYC-H, and Q10 groups (*P<0.05* or *P<0.01*) (**Figures 3A, B**).

To investigate the level of OS, the amount of SOD, GSH-Px, MDA, and T-AOC in serum were determined. There was a significant increase in SOD and GSH-Px levels in the CTX group compared to the CON group (*P<0.01*). Conversely, the MDA level was higher than that in the CON group (*P<0.01*). Compared to the CTX group, SOD and GSH-Px were increased in HSYC-H, HSYC-M and Q10 groups, while MDA decreased (*P<*0.05 or *P<*0.01). Additionally, T-AOC exhibited a significant decrease in the CTX group compared with that in the CON group (*P<0.01*), and was considerably elevated in the HSYC-M, HSYC-H, and Q10 groups compared to the CTX group (*P<0.05* or *P<0.01*) (**Figures 3C-F**).

### 3.4 HSYC protected against mitochondrial dysfunction in GCs of POI afflicted mice

**Figure 4:**
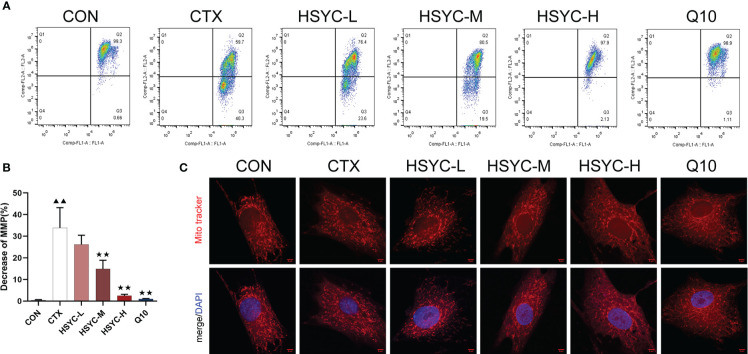
HSYC improved mitochondrial dysfunction in ovarian granulosa cells of POI afflicted mice. **(A)** Flow cytometric analysis of mitochondrial membrane potentials degradation, Q2 represents granulosa cells with normal mitochondrial membrane potentials and Q3 represents granulosa cells with decreased mitochondrial membrane potentials. **(B)** The histogram plots means with SD of flow cytometric analysis of mitochondrial membrane potentials degradation. Histogram elements represent the mean ± SD (n=3). ^▲▲^ presents *P < 0.01* compared to the CON group; ^★★^presents *P < 0.01* compared to the CTX group. **(C)**. Observation of mitochondria morphology by MitoTracker Red staining. Red color represents the mitochondria and blue represents nucleus of granulosa cells. Scale Bar: 5µm.

To explore the protective effect of HSYC on mitochondrial dysfunction, we investigated the MMP level in GCs by flow cytometry using JC-1 and mitochondrial staining. Q2 represented cells with normal mitochondrial membrane potential, while Q3 represented cells with decayed mitochondrial membrane potential. Compared with the CON group, the mitochondrial membrane potential reduction rate of GCs in the CTX group was significantly increased (*P<0.01*). However, in the HSYC-M, HSYC-H, and Q10 groups the mitochondrial membrane reduction rate was decreased (*P<0.01*), indicating that the cellular mitochondrial membrane potential was considerably elevated (**Figures 4A, B**). Similarly, mitochondria of GCs in the CON group exhibited generally tubular-reticular morphology, as confirmed by mitochondrial staining analyses, and the CTX group exhibited spherical mitochondria. Compared with the CTX group, mitochondria were longer and displayed a reticular distribution in each treatment group (**Figure 4C**).

### 3.5 HSYC improved mitophagy in GCs of POI afflicted mice

**Figure 5:**
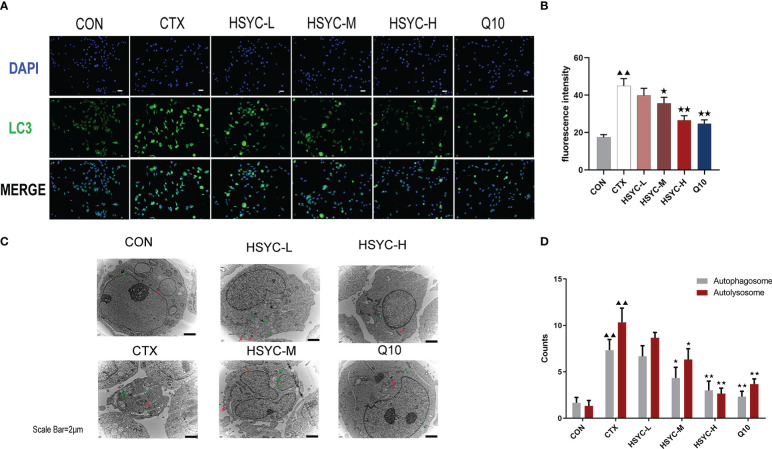
HSYC improved autophagy in GCs of POI afflicted mice. **(A, B)** Immunofluorescence images of the LC3. Green color represents the LC3 and blue represents nucleus of granulosa cells. Scale bar: 25μm. The result is shown using histogram plots means with SDs represent LC3 fluorescent intensity levels. **(C, D)**. Observation of autophagosomes and autophagolysosomes in granulosa cells of different groups under transmission electron microscopy. Scale bar:2μm. The result is shown using histogram plots means with SDs represent autophagosome and autophagolysosome counts. Histogram elements represent the mean ± SD (n=3). ^▲▲^ presents *P < 0.01* compared to the CON group; ^★^presents *P < 0.05* compared to the CTX group; ^★★^presents *P < 0.01* compared to the CTX group.

The quantification data of LC3 fluorescent intensity indicated that expression levels in the GCs of the CTX group were significantly higher than those in the CON group (*P<0.01*). The LC3 expression levels in the HSYC-M and HSYC-H groups were lower than those in the CTX group (*P<0.05* or *P<0.01*). A similar trend was observed in the Q10 group. (**Figures 5A, B**)

To evaluate the autophagy level, we observed the ultrastructure of autophagosomes and autophagolysosomes of GCs under TEM. The GCs in the CON group had clear nuclei and abundant organelles, such as endoplasmic reticulum and mitochondria. Autophagosomes and autophagolysosomes were present in lesser numbers. Conversely, there was a sharp increase in the number of those in the CTX group (*P<0.01*). HSYC-M, HSYC-H, or Q10 treatment led to a considerable reduction in the number of autophagosomes and autophagolysosomes compared with the CTX group (*P<0.05* or *P<0.01*), which indicated an improvement in the degree of autophagy (**Figures 5C, D**).

**Figure 6:**
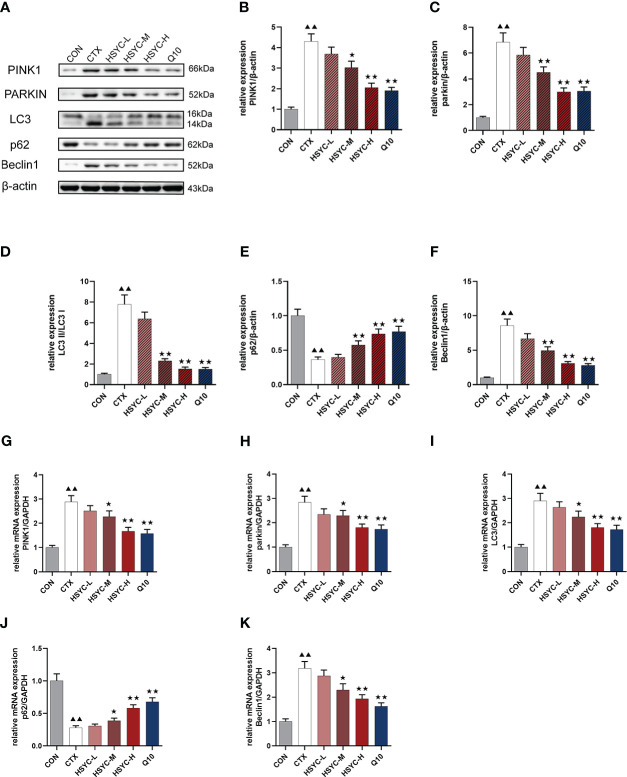
Effect of HSYC on the expression of mitophagy-associated genes and proteins. **(A-F)** Effect on PINK1, Parkin, LC3, Beclin1 and p62 proteins in the granulosa cells of different groups. **(G-K)** Effect on PINK1, Parkin, LC3, Beclin1 and p62 mRNAs in the granulosa cells of different groups. Histogram elements represent the mean ± SD (n=3). ^▲▲^ presents *P < 0.01* compared to the CON group; ^★^presents *P < 0.05* compared to the CTX group; ^★★^presents *P < 0.01* compared to the CTX group.

We assessed the mRNA and protein expression of mitophagy-related genes using qPCR and WB, respectively. In the CTX group, the mRNA and protein expression levels of PINK1, Parkin, LC3, and Beclin1 were higher, whereas p62 was lower compared with the CON group (*P<0.01*). With HSYC-M, HSYC-H, or Q10 intervention, the expression levels of PINK1, Parkin, LC3, and Beclin1 were significantly decreased, and p62 were significantly increased compared with the CTX groups (*P<0.05* or *P<0.01*) (**Figure 6**). The original bands are presented in **Supplementary Figures S2–7**.

### 3.6 HSYC downregulated the NLRP3 inflammasome in GCs of POI afflicted mice

**Figure 7:**
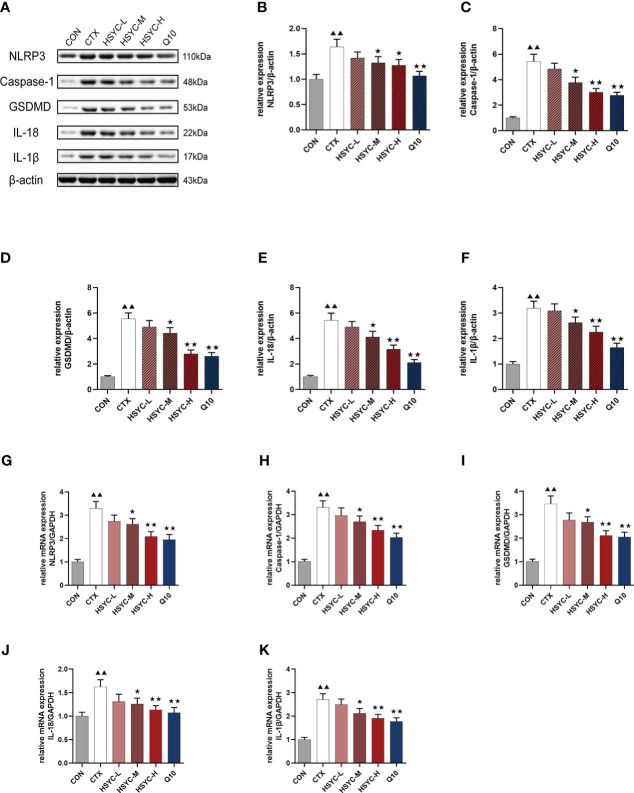
Effect of HSYC on the expression of NLRP3 inflammasomes-associated genes and proteins. **(A-F)** Effect on NLRP3, Caspase-1, GSDMD, IL-18, and IL-1β proteins in the granulosa cells of different groups. **(G-K)** Effect NLRP3, Caspase-1, GSDMD, IL-18, and IL-1β mRNAs in the granulosa cells of different groups. Histogram elements represent the mean ± SD (n=3). ^▲▲^ presents *P < 0.01* compared to the CON group; ^★^presents *P < 0.05* compared to the CTX group; ^★★^presents *P < 0.01* compared to the CTX group.

mRNA and protein expression levels of NLRP3 inflammasome were detected by qPCR and WB analyses, respectively. Results indicated that the NLRP3, caspase-1, GSDMD, IL-18, and IL-1β mRNA expression in the CTX group was significantly higher than that of the CON group (*P<0.01*). Compared with the CTX group, NLRP3, caspase-1, GSDMD, IL-18, and IL-1β mRNA expression was significantly lower in the HSYC-M, HSYC-H, and Q10 treatment groups (*P<0.05* or *P<0.01*). WB analyses showed similar results (**Figure 7**). The original bands are presented in **Supplementary Figure S2, 8-12**.

## 4 Discussion

POI is a common gynecological disease that affects ovarian endocrinological function and reduces pregnancy rates (23). To date, modern medical treatments are limited and do not fundamentally improve ovarian function (24), whereas TCM has been proven to be an important adjunct. HSYC nourishes yin, tonifies the kidney, and has been widely used in clinical practice for treating POI for almost a hundred years. In this study, the results showed that HSYC can improve ovarian endocrine function and increase the number of follicles in a chemotherapy-induced POI model. Similarly, another study performed by our team found that HSYC can decrease the proportions of fragmented oocytes to enhance oocyte quality (8), therefore, confirming the therapeutic effect of HSYC for POI.

We evaluated the effect of HSYC on OS in a POI model. ROS are oxygen-containing reactive chemical species which engage in cell signaling and promote cell survival, proliferation, and differentiation at the physiological level (25). Once ROS levels exceed cellular antioxidant levels, they can react with DNA, proteins, lipids, and carbohydrates; causing DNA strand breaks and protein and lipid oxidation (26). Ovarian function regulations, such as folliculogenesis and steroidogenesis, have been proven to be related to the generation of ROS in female reproductive organs (27). As one of the products of lipid peroxidation, MDA levels are also indicative of the degree of oxidative injury (28). In contrast, SOD and GSH-Px are essential antioxidant components in cells and play a crucial role in maintaining redox equilibrium (29). Our study found that oxidative stress markers were higher and antioxidants were reduced in the POI model, and this effect was reversed by HSYC or Q10 treatment to some extent. As a component of the electron transport chain, Q10 is a kind of important antioxidant in mitochondria which is extensively used to improve ovarian reserve (30). Our result further demonstrated that HSYC had an effect on protecting against oxidative damage generated by CTX in ovarian tissues closed to that of Q10.

Mitochondria are the main source and target of ROS and play the most critical role in OS. There is a vicious cycle that amplifies OS and damages mitochondrial ultrastructure, exacerbating mitochondrial dysfunction, which causes the continued production of ROS (31). It is shown in many studies that an excessive buildup of ROS can do damage to oocytes and GCs, which in turn cause ovarian senescence and reduced ovarian reserve (11, 14, 32).Similarly, our result showed that ROS levels were increased in the POI group. Mitochondrial ATP generation is critically valuable in oocytes and early mammalian embryos (33).Meanwhile, MMP is a biomarker of mitochondrial activity that is vital in ATP synthesis, redox balance, signaling, and metabolism (34, 35). Recent studies have found that lower MMP and ATP levels are closely related to decreased mitochondrial function in GCs (36).

In line with the above conclusion, our findings suggests that the insufficiency functions of granulosa cell in POI was related to elevated ROS level, decreased MMP and ATP, which indicated considerable mitochondrial dysfunction. In addition, the results showed both HSYC and Q10 can ameliorate mitochondrial damage and improve mitochondrial function. This is the first study revealing the similar antioxidative effect of HSYC compared to Q10.

To maintain a healthy mitochondrial population, damaged mitochondria are selectively degraded by mitophagy (37), therefore, it is also an important indicator for evaluating mitochondrial function. To induce mitophagy, PINK accumulates at the outer membranes of damaged mitochondria, where it recruits and activates Parkin, an E3-ubiquitin ligase, triggered by recruitment to the surface of injured mitochondria, causing ubiquitination of mitochondrial surface proteins, accumulation of substrate binding receptors, and subsequent binding to LC3, initiating autophagy (38). Parkin is regulated by upstream PINK1, both of which are essential proteins for mitophagy. Autophagy-related genes (ATG)8 is the mammalian equivalent of LC3. Upon activation of mitophagy, LC3-I is transformed to LC3-II and localized to the autophagosomal membrane. This membrane can be destroyed after autophagic vesicles bind to lysosomes and form autophagic lysosomes (39). Consequently, LC3 expression can indicate the amount of autophagic vesicle activation. P62 is one of the most significant ubiquitinated substrates. Ubiquitinated substrates are essential to all autophagic activities. When mitophagy is activated, it can attach to ubiquitinated proteins to produce p62 aggregates which then recognize and bind to LC3-II in the autophagosomal endosome; enabling further expansion of the autophagosome and enveloping the substrate, which is ultimately destroyed by the lysosome (40). P62 expression is negatively correlated with the level of mitophagy (41). Beclin-1 is an ATG6 homologous protein that forms a complex with class III phosphatidylinositol 3-kinase (PI3K III) and ATG14 in the production of autophagic vesicles. The level of Beclin-1 protein expression is strongly linked with mitophagy activation (42).

Previous studies have proved that PINK1/Parkin-mediated mitophagy is activated by overexpressed ROS inside the mitochondria (43). Meanwhile, mitophagy of GCs is associated with ovarian dysfunction (44). Consistent to these results, we found increased mitophagy in the ovaries of the model group.

Remarkably, this results further revealed that the mitophagy of GCs maybe *via* Pink1/Parkin pathway, which is attributable to the mitochondrial damage caused by CTX and the excessive accumulation of ROS. Furthermore, HSYC significantly reduced mitochondrial dysfunction in POI mice, and therefore mitophagy to remove damaged mitochondria was also significantly reduced.

Notably, further analysis of our results demonstrated that NLRP3 inflammasome activation was associated with POI. Several published studies have additionally revealed that mitochondrial dysfunction and high mitochondrial ROS are essential for NLRP3 activation (45, 46). Furthermore, the mitochondrial membrane serves as a platform for the assembly of the inflammasome and the inflammatory response (47, 48). Consistent with these prior findings, our research discovered that CTX-induced mitochondrial dysfunction may initiate the NLRP3 inflammasome-pyroptosis cascade. In contrast, HSYC may improve ovarian reserve function and decrease the progression of POI by reducing mitochondrial damage and blocking pyroptosis. Along with the previously known GC apoptosis, our study demonstrated for the first time that CTX-induced ovarian impairment was also mediated by cell pyroptosis. However, our study had some limitations including a lack of *in vitro* validation experiments to confirm the relationship between mitophagy and pyroptosis in POI, and we aim to explore this in future studies.

In contrast to the “one drug for one target” mode of modern medicine, multipurpose and multitarget medicines are the hallmark of TCM (49). Our investigation revealed two significant conclusions. First, HSYC enhanced ovarian function from CTX-induced ovarian damage and OS. Second, HSYC protected GCs in mice with POI by inhibiting PINK1-Parkin mitophagy and NLRP3 inflammasome activation. Our research provides scientific evidence for the treatment of POI with HSYC and suggests a potential future therapeutic application.

## Data availability statement

The raw data supporting the conclusions of this article will be made available by the authors, without undue reservation.

## Ethics statement

The animal study was reviewed and approved by Institutional Animal Care, the Animal Experiment Center of Zhejiang Chinese Medical University.

## Author contributions

CM, YZ and YC designed and performed most experiments, and wrote the manuscript. RW analyzed the data. NR helped preform the animal experiments. BC helped analyze the flow cytometry. PD edited the manuscript. QZ supervised all experiments, and edited the manuscript. All authors contributed to the article and approved the submitted version.

## Supplementary material

Supplementary Figure 1 Identification of the isolated GCs. **(A)** Morphological changes of granulosa cells cultured for 1-6 days under light microscope. Scale bar: 100um. **(B)** Isolated granulosa cells with H&E staining. Scale bar: 40um.

Supplementary Figure 2 The original bands for β-actin.

Supplementary Figure 3 The original bands for Beclin 1.

Supplementary Figure 4 The original bands for p62.

Supplementary Figure 5 The original bands for parkin.

Supplementary Figure 6 The original bands for PINK1.

Supplementary Figure 7 The original bands for LC3.

Supplementary Figure 8 The original bands for NLRP3.

Supplementary Figure 9 The original bands for GSDMD.

Supplementary Figure 10 The original bands for caspase-1.

Supplementary Figure 11 The original bands for IL-18.

Supplementary Figure 12 The original bands for IL-1β.
