# Supplementary material for: Investigation of He’s Yang Chao recipe against oxidative stress-related mitophagy and pyroptosis to improve ovarian function
Source: Front Endocrinol (Lausanne). 2023 Jan 27;14:1077315. doi: 10.3389/fendo.2023.1077315 (PMC9911881; doi:10.3389/fendo.2023.1077315)
Supplement: Supplementary Figure 1 — Identification of the isolated GCs. (A) Morphological changes of granulosa cells cultured for 1-6 days under light microscope. Scale bar: 100um. (B) Isolated granulosa cells with H&E staining. Scale bar: 40um. [file DataSheet_1.docx]

Supplementary Material

## Supplementary Figures

**
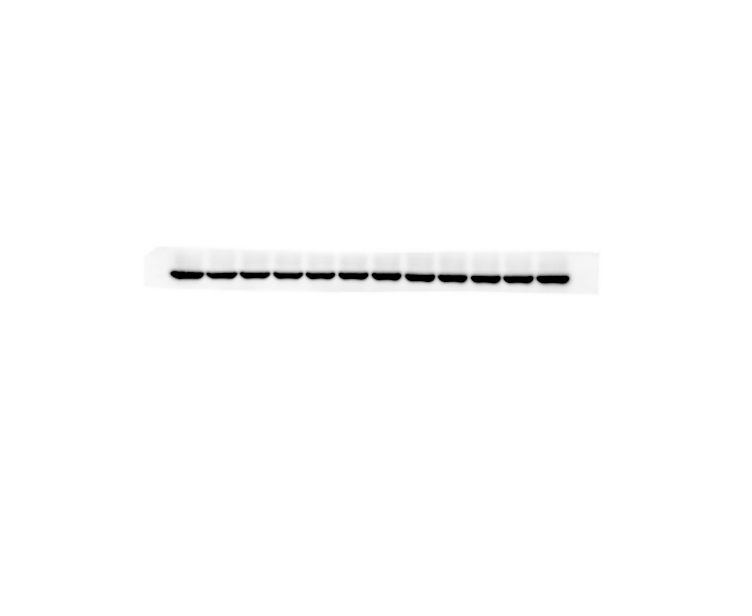
**

S2: The original blot for the cropped WB band of βactin.
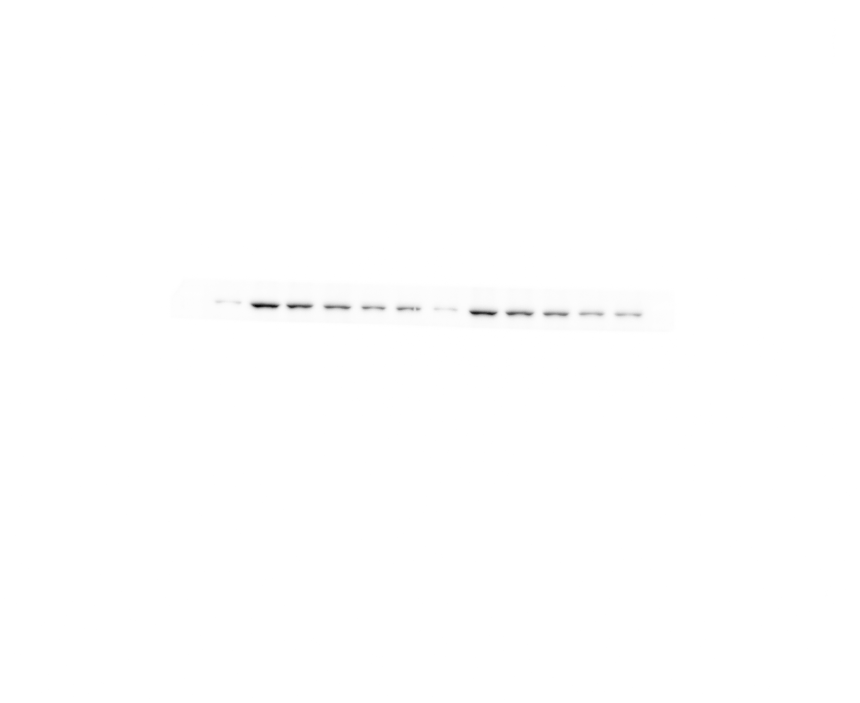


S3: The original blot for the cropped WB band of Beclin1.


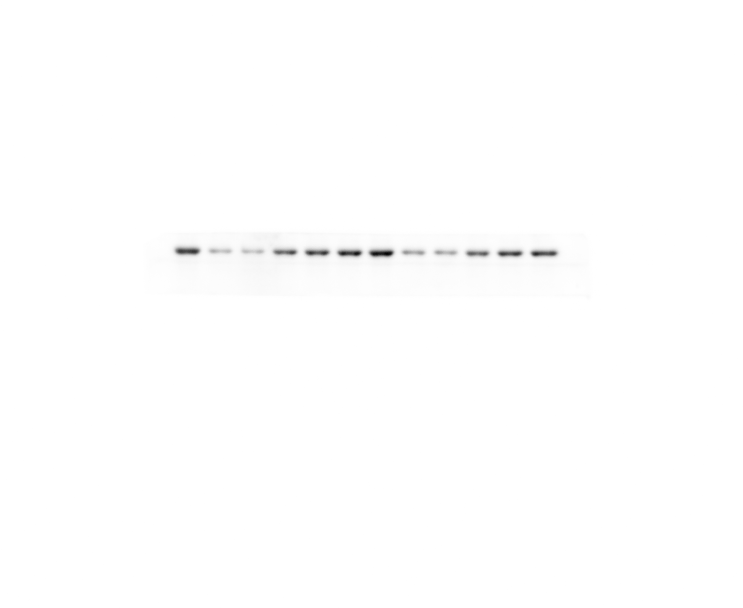


S4: The original blot for the cropped WB band of p62.


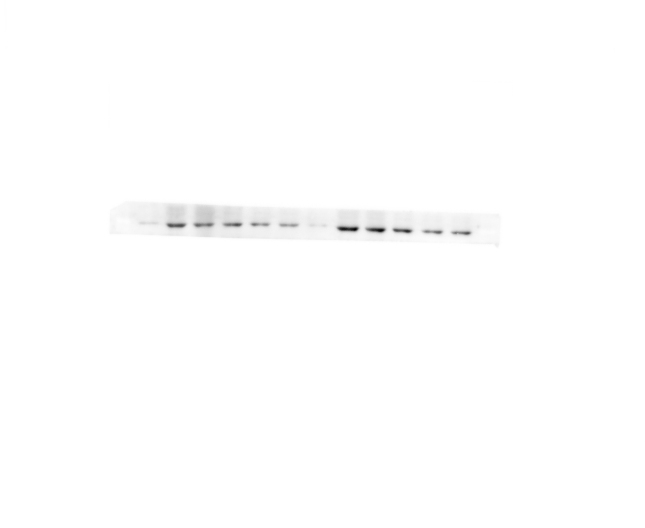


S5: The original blot for the cropped WB band of parkin.


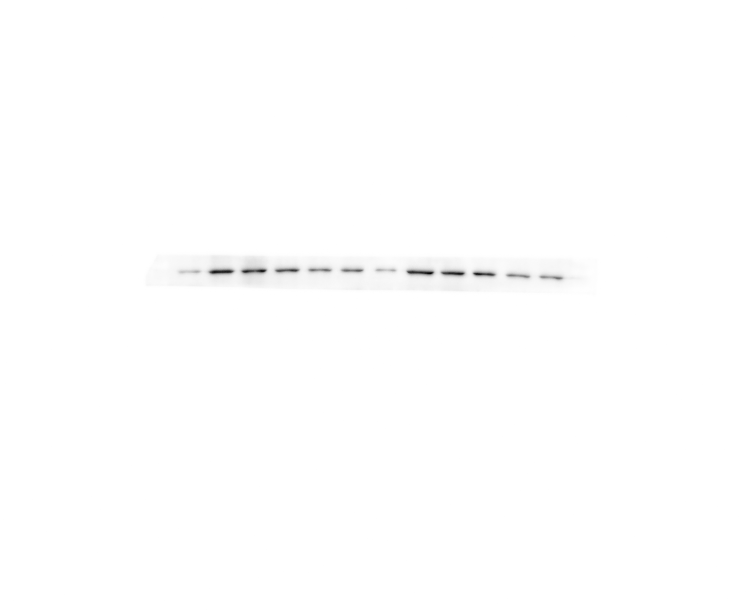


S6: The original blot for the cropped WB band of PINK1.


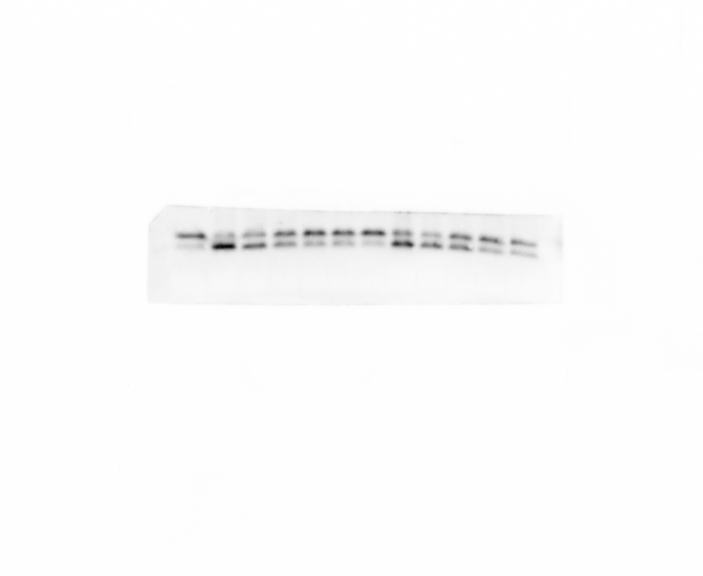


S7: The original blot for the cropped WB band of LC3.


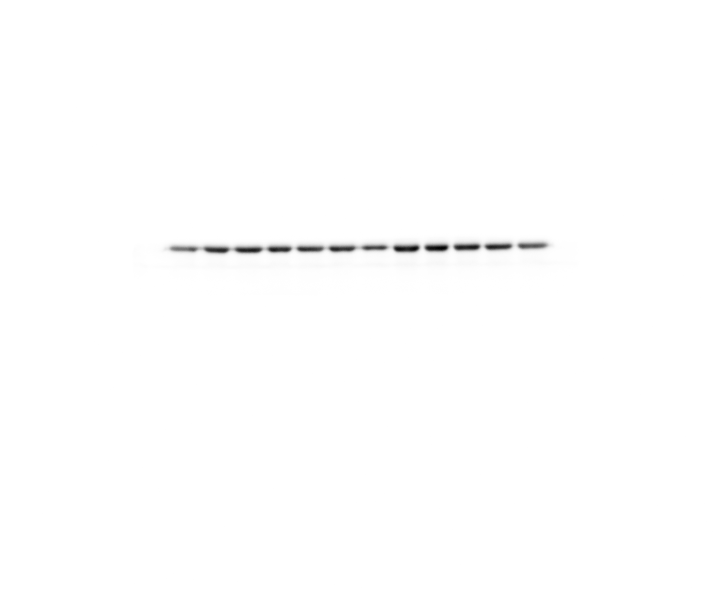


S8: The original blot for the cropped WB band of NLRP3.


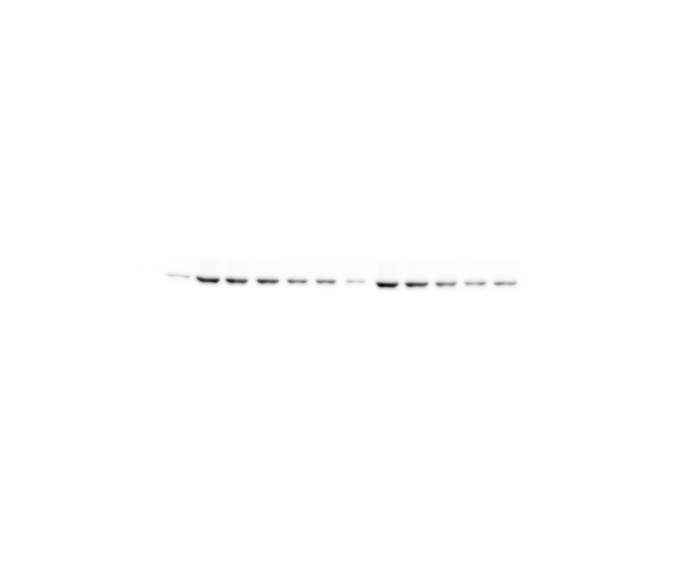


S9: The original blot for the cropped WB band of GSDMD.


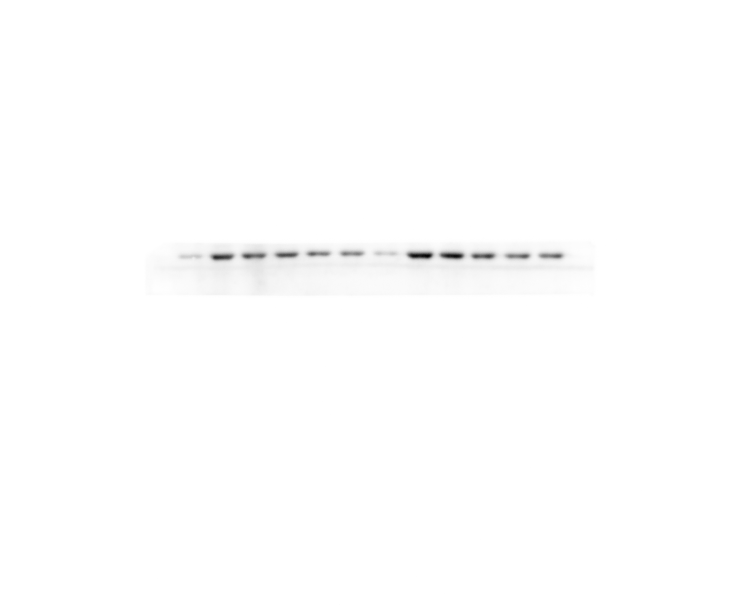


S10: The original blot for the cropped WB band of caspase1.


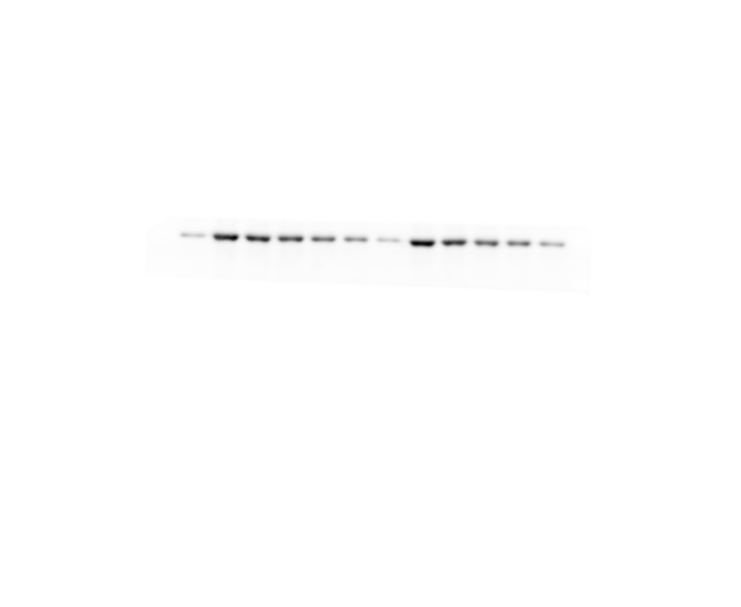


S11: The original blot for the cropped WB band of IL-18.


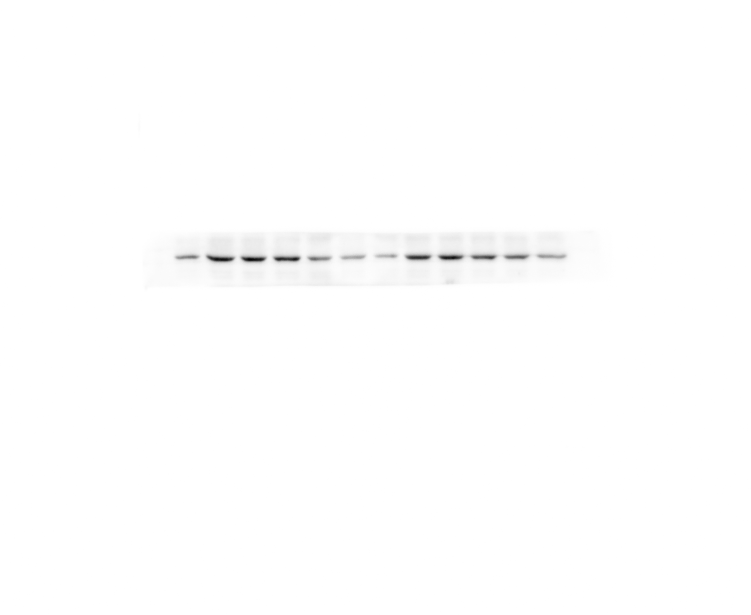


S12: The original blot for the cropped WB band of IL-1β.
