# Supplementary figures and images for: Investigation of He’s Yang Chao recipe against oxidative stress-related mitophagy and pyroptosis to improve ovarian function
Source: Front Endocrinol (Lausanne). 2023 Jan 27;14:1077315. doi: 10.3389/fendo.2023.1077315 (PMC9911881; doi:10.3389/fendo.2023.1077315)

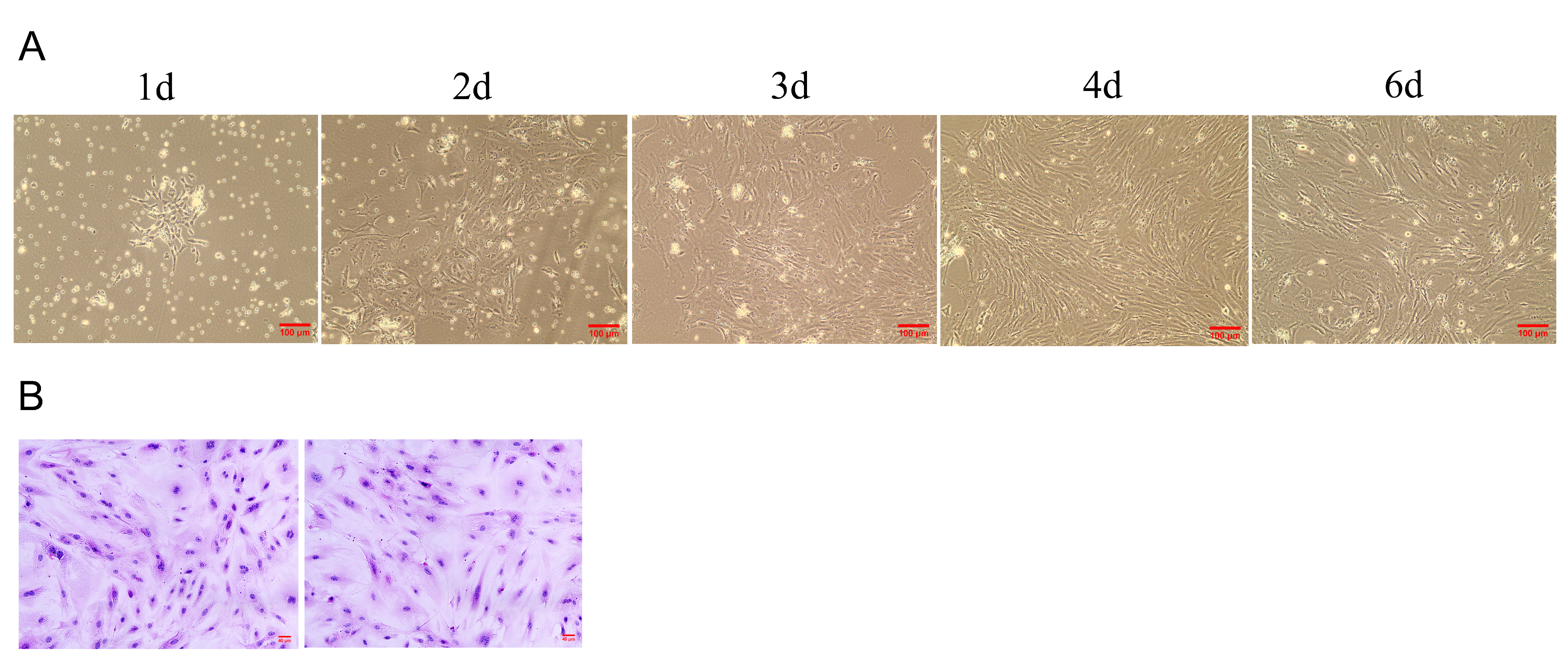

Supplement: Supplementary Figure 12 — The original bands for IL-1β. [file Image_1.tif]
